# From dusk to dawn: examining how adolescents engage with digital media using objective measures of screen time in a repeated measures study

**DOI:** 10.1186/s12966-024-01698-0

**Published:** 2025-01-07

**Authors:** Bradley Brosnan, Kim A. Meredith-Jones, Jillian J. Haszard, Shay-Ruby Wickham, Barbara C. Galland, Takiwai Russell-Camp, Rachael W. Taylor

**Affiliations:** 1 Department of Medicine, University of Otago, PO Box 56, Dunedin, 9010 New Zealand; 2 Hazard Biostatistics, Kaka Point, New Zealand; 3 Department of Women’s and Children’s Health, University of Otago, Dunedin, New Zealand

**Keywords:** Screen time, Digital device, Youth, Sleep, Accelerometry, Video analysis

## Abstract

**Background:**

Although evening screen time is thought to impair subsequent sleep, current measures are limited to questionnaires which seem unlikely to accurately assess screen time in youth. Given the ubiquitous nature of digital devices, improving measurement of screen time is required before related health effects can be appropriately determined. The aim of this study was to objectively quantify screen time before sleep using video camera footage.

**Methods:**

This repeated-measures observational study in healthy adolescents (11–14 years) from Dunedin, New Zealand measured screen time on four evenings over one week in the home environment from March-December 2021. Wearable and stationary PatrolEyes video cameras captured screen time from two hours before bedtime until sleep and manually coded for device type (phone, tablet, laptop computer, desktop computer, handheld gaming console, gaming console, television and other) and screen activity (watching, listening, reading, educational/creative, browsing, communication, social media, video gaming, multitasking) using a reliable coding schedule (κ ≥ 0.8). Descriptive findings are reported.

**Findings:**

Among the 83 participants (mean 12.3 [SD 1.0] years, 42% female, 52% New Zealand European, 37% Māori [indigenous]), 82 used screens in the two hours before bed on 308 of 344 (90%) nights for a mean of 54.4 min (SD 25.5). Televisions (median 37 min, 56% of nights), phones (19 min, 64% nights), and multitasking using multiple devices (19 min, 48% nights) were most commonly used (> 75% of adolescents). Once in bed but before trying to sleep, 58% of adolescents engaged in screen time for 17 (26.3) minutes on 36% of nights. The most common screen activities were watching (32.5%), social media (26.5%) and communication (20.5%). Even after attempting sleep, 32.5% of participants used screens for 8.0 min (median) on 16% of nights, mostly listening on phones.

**Conclusions:**

Objective video cameras offer detailed insight into evening screen habits, capturing frequency, content, and duration. Youth frequently engage with screens before bed and throughout the night on a range of activities, despite recommendations to restrict screen time prior to sleep.

**Trial registration:**

Australian New Zealand Clinical Trials Registry (anzctr.org.au), AACTRN12621000193875, Registered 23 February 2021, https://www.anzctr.org.au/Trial/Registration/TrialReview.aspx?id=380926&isReview=true.

**Supplementary Information:**

The online version contains supplementary material available at 10.1186/s12966-024-01698-0.

## Background

Widespread use of digital devices has made screen time a fundamental part of modern life, especially for youth [1], leading to concerns about potential impacts on health and wellbeing, including sleep [2, 3]. However, most previous research has relied on self- or parent-reported measures of screen time, which are prone to recall bias and fail to provide a comprehensive understanding of actual screen usage [4, 5]. Most research has also been restricted to simple estimates of total daily screen time or a limited range of device types (e.g., mobile phones versus television) or activities (e.g. social media) which seems unlikely to provide adequate assessment in today’s world [6–9]. Improving our understanding of other complex screen behaviours, such as multitasking, where more than one device is used simultaneously, is of considerable interest, but difficult to measure [10, 11].

A variety of software and application logging tools are available that objectively measure screen use. While these tools offer promise for accurately determining screen time on specific devices (e.g., Android phones) [12], they do not capture interactions and usage across all types of digital devices [13, 14]. By contrast, wearable photo cameras that are worn on the body facing outwards are able to capture where, when and how individuals use screens by taking static images at fixed intervals ranging from 5 to 30 s [15], and can thus objectively measure screen time across multiple devices [16]. However, wearable photo cameras cannot capture sound, motion or speed, which may be important for understanding all aspects of screen time. It is also not clear how much screen time might be ‘missed’ given photos are only taken at intervals. By contrast, video cameras provide continuous recording and could offer a comprehensive understanding of usage patterns, including quick switching usage and contextual information [17]. To date, no studies appear to have examined screen time in adolescents assessed using video camera footage.

We developed a reliable and comprehensive protocol for coding screen use from video camera footage in terms of timing, duration, device type (eight categories) and screen activity (nine categories) in adolescents [17], including the ability to measure multitasking within the same device (e.g. watching a movie on YouTube minimised while also scrolling through social media) and across different devices (e.g. gaming on an Xbox while also watching a movie on a laptop). We used this coding protocol to objectively measure evening screen time in children and adolescents as part of the Bedtime Electronic Devices (BED) study. The aim of this analysis was to objectively quantify type and duration of screen time before sleep using video camera footage in 11 to 14 year olds.

## Methods

### Study design

BED was a repeated-measures observational study investigating evening screen time on four non-consecutive nights over the course of one week in adolescents aged 11 to < 15 years. The primary outcome was the effect of screen time on sleep that night [18], but data were also collected on dietary intake and wellbeing the next day which will be reported elsewhere. As such, participants were informed that BED aimed to observe a range of health behaviours, including diet, physical activity and screen use. BED had ethical approval from the University of Otago Human Ethics Committee (H20/065) and was registered with the Australian New Zealand Clinical Trials Registry (anzctr.org.au, AACTRN12621000193875, 23 February 2021). Eligible participants (11 to < 15 years of age, lived locally, no physical or mental barriers to participation) were recruited by advertisement (mainly via social media) and word of mouth from March to December 2021. Written informed consent was obtained from both parents and adolescents before data collection commenced.

### Sample size

A sample size of 66 participants was required for the primary outcome to reliably estimate the relationship between pre-bedtime screen behaviours and sleep duration and quality, assuming no more than four predictors in the model, a relatively high intra-class correlation of 0.7, and at least three nights of sleep [18]. We aimed to recruit 85 participants to allow for missing data and drop-outs.

### Outcomes

Parents completed a brief questionnaire regarding demographic characteristics, including their highest education level and the ethnicity of the adolescent. In New Zealand, ethnicity is self- (or parent-) identified, and multiple ethnicities can be selected. A prioritisation system is then applied, in order of Māori (indigenous New Zealanders), Pacific, Asian, and New Zealand European and others. A brief questionnaire at study exit assessed potential reactivity to the presence of the cameras. Participants were asked if they felt that wearing the camera had changed their screen behaviours in any way (answer options of ‘no, I did everything I normally would’, ‘yes, some things differently’, or ‘yes, lots of things differently’). A free text box to explain further was also provided. Informal conversations were also undertaken with most participants around this issue, but have not been reported here.

Over four non-consecutive nights across one week, participants wore a PatrolEyes SC-DV7 Ultra video camera (PatrolEyes, Ada, Michigan, USA) on a chest harness (facing outwards) from three hours before bedtime (to increase the likelihood of collecting data from two hours before bedtime for all participants) until they went to bed (wearable camera). A second identical camera was mounted on a tripod in their bedroom and recorded any screen use after the participant had gone to bed until awakening in the morning (stationary camera). Participants were asked to turn these cameras on 30 minutes before bedtime to prevent gaps in data footage. These compact (7.7 × 5.6 × 2.8 cm, weight 128 g) video cameras record continuous video footage at high resolution (1080p) via a wide-angle lens with a 170° field of vision; the infrared night vision mode captures screen usage after ‘lights out’. The BED study adhered to ethical frameworks for using wearable cameras in health behaviour research [19], including providing adolescents and families with substantial information on how to safeguard their (and others’) safety and privacy, and allowing participants and families to view all footage first and delete any that they did not want us to see.

The videos were downloaded to a secure, high-capacity university storage system and erased from the cameras. Video footage was coded using an established reliable protocol [17] that quantified when and for how long participants spent time on eight different devices (phone, tablet, laptop computer, desktop computer, handheld gaming console, gaming console, television, other) and nine screen activities (watching, listening, reading, educational/creative, browsing the internet, communication, social media, video gaming, multitasking within a device). Data regarding multitasking across multiple devices was also coded. The nine screen activities were also collapsed into passive (watching, listening, reading, browsing, other unknown passive) and interactive (gaming, communication, device-based multitasking, educational or creative tasks, other unknown interactive) activities. Social media was excluded from these broader categories, being a blend of both passive and interactive screen usage. Complete descriptions of each category have been previously reported including examples of the images obtained [17].

### Video coding

Four researchers coded 1081 h of footage using Observer XT version 16.0 (Noldus Information Technology Inc., Leesburg, VA, USA) on a second-by-second basis. To qualify as a new code (indicating a change in screen behaviour), the participant needed to exhibit the new behaviour for at least three seconds. This time frame was determined for practical purposes and ease of coding given the vast amount of video footage obtained. Reliability statistics between different coders were calculated within Noldus Observer XT for frequency (compares the order in which annotations or changes in screen behaviour occur) and sequence (also examines the time when each annotation or change in screen behaviour occurs so is a more conservative analysis) analyses. Inter-rater reliability (average weighted kappa [κ]) was determined from each coder independently coding a random subset of 83 30-minute video files. Drift reliability was calculated from coders re-coding a random subset of 21 video files approximately one month after initial coding [17]. Adequate inter-rater reliability was indicated by per cent agreement of ≥ 90% and average weighted κ of at least 0.80 [20].

Screen use was examined for three specific time periods; two hours before bed, while in bed but before attempting to sleep, and after shuteye time (Supplementary Fig. 1). Bedtime was identified from the video footage as when the participant went to bed for the night and got underneath the bed covers. Shuteye time was identified as when the participant was in bed, had stopped all interactions (including any screen time and conversations with parents) and was attempting to go to sleep [21]. If video footage for bedtime or shuteye times was absent, subjective daily diary entries (*n* = 14 nights) or a questionnaire assessing ‘usual’ bedtime and shuteye times (*n* = 2) was used.

### Statistical analysis

Stata version 17.0 (StataCorp LLC) was used for the descriptive analyses following export of the video data in one second intervals for each coded video for each day of data using Noldus Observer XT. Data were aggregated for all days of data and summarised into variables of interest, including total screen time, time on specific devices, and time doing different screen activities, for each time period (two hours before bed, between bedtime and shuteye time, and after shuteye time). Because night-by-night screen use differed, overall means and standard deviations (SD) were reported for the whole sample, whereas medians and 25th and 75th percentiles were used for nights when screens were used. Within-person standard deviations were also reported to describe variability across the week. Total screen time was calculated by summing the duration of any screen use. This included times when either a single screen or multiple screens were in use concurrently (i.e. ‘multitasking’), which ensured that the total screen time was not inflated due to simultaneous multi-screen activity. Periods of missing footage were assumed not to represent screen time based on the study protocol, which stipulated that participants would not record if they were outside the house, in the bathroom, showering or bathing [17]. Footage that was ‘blocked’ or had an obscured view was considered inconclusive for determining screen behaviour, especially if the camera angle was directed away from the screen, there was no device audio, or the image was blackened due to obstruction. If a device type was known to be in use but the screen activity was blocked, the time was still coded as a device type but not as a specific activity.

## Results

**Fig. 1:**
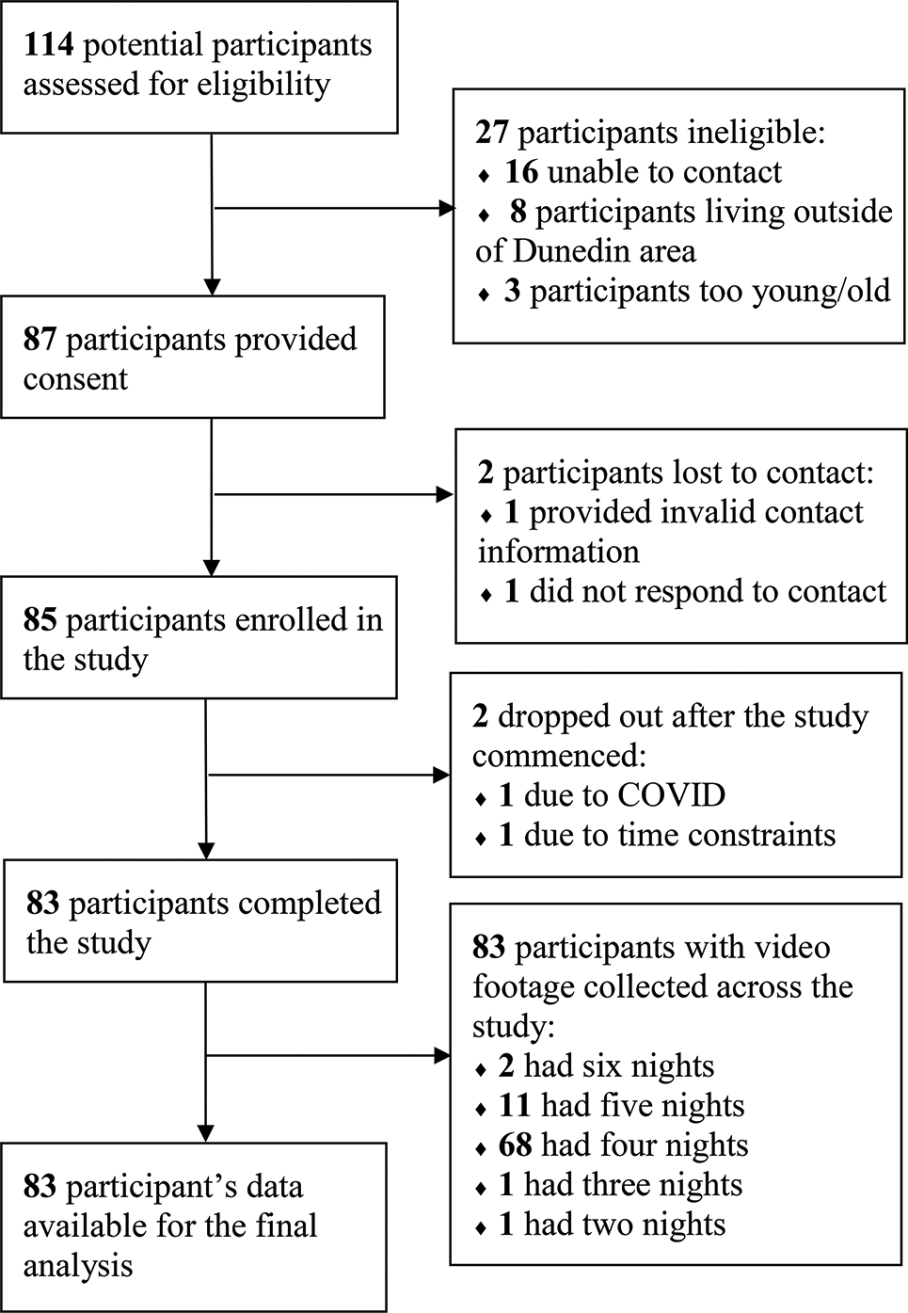
CONSORT diagram

Of the original 85 participants, two withdrew (COVID-19 lockdown, time constraints) leaving 83 for analyses (Fig. 1). These 83 participants were 12.4 (1.0) years of age and 42% were female. While the proportion of indigenous Māori youth was high at 37%, parental education was also high (48% University educated) and socioeconomic deprivation relatively low (Table 1).

**Table 1:** Demographic characteristics of participants (*n* = 83)

Variables	Total (%)
Age, mean (SD) years	12.4 (1)
Sex, n (%) female	35 (42)
Ethnicity, n (%)	
New Zealand European	43 (52)
Māori (indigenous population of New Zealand)	31 (37)
Pacific	4 (5)
Asian	5 (6)
Parental education n (%)	
High school	10 (12)
Polytechnic or similar tertiary qualification	33 (40)
University educated	40 (48)
Socioeconomic deprivation^1^	
High (NZ Dep 8–10)	17 (21)
Medium (NZ Dep 4–7)	34 (41)
Low (NZ Dep 1–3)	32 (39)
Sleeping environment	
Shares a room with someone else (%)	20 (24)

^1^ Determined using the New Zealand Index of Deprivation 2018 [31] which combines 9 variables from the 2018 New Zealand national census to determine a deprivation score which estimates the relative material and social deprivation for the area where the participant lives, where decile 1 represents areas with the least deprivation and decile 10 represents areas with the most deprivation

Each participant contributed around 13 h of coded footage across all nights, with 344 nights in total. In total, just 372 min of footage was deleted by participants. Median (25th, 75th percentiles) camera footage was 89 (68, 107) minutes in the two hours before bedtime, and 20 (9, 42) minutes between bedtime (median 9:33 pm) and shuteye time (median 9:58 pm) producing a shuteye latency of 22 (8, 46) minutes (Supplementary Table 1). The time frame after shuteye time (i.e. until morning wake) was not coded if no screens were in use. Inter-rater reliability was calculated as a weighted κ value of 0.92 (SD 0.1) and drift reliability as κ = 0.80 (SD 0.1).

### Screen time in the two hours before bed

Table 2 demonstrates that all but one participant had screen time in the two hours before bed across 89.5% of the nights, for a median duration of 56.4 min. However, individual variability was high as indicated by a mean within-person standard deviation of 27.5 min. Phones (86.8% of participants) and television (84.4%) were the most commonly used devices, but multitasking across multiple devices was also very high (75.9%). On the nights when devices were used, youth spent a median of 19.0 (25th, 75th percentiles: 7.2, 31.8) minutes on their phones, 37.2 (12.8, 63.1) minutes watching television, and 18.8 (8.9, 37.9) minutes multitasking. While the median minutes spent on gaming consoles was relatively high (30.2 min), only about one-third of youth used this device on just 17% of nights. In terms of specific screen activities, watching was most prevalent, with 91.6% of participants watching something on 71.5% of nights for a median duration of 38.1 (22.1, 59.2) minutes. Browsing (81.9% participants, 43.9% nights) and communication (78.3% participants, 43.6% nights) were also common, but only for short durations (median of 3.7 min each). Many adolescents (61.5%) spent time on social media (33.1% nights) but only for 7.8 (2.1, 19.4) minutes during this pre-bedtime period. Adolescents spent little time listening, reading or undertaking educational or creative activities and around 10 min per night could not be coded (blocked/not in view, Table 2). Exploratory analyses showed relatively few sex differences in usage, with the exception of gaming consoles and multitasking appearing more common in males and social media more common in females in the pre-bed period (Supplementary Table 2).

**Table 2:** Screen time in the two hours before bed across device and activity types for 83 participants over a possible 344 nights

	Overall mean duration min (SD)	Number (%) of participants who had screen time	Total number (%) of nights with screen time	Median (25th, 75th percentile) duration when used, min	Mean (SD) within-person SD of screen time min
Total screen time	54·4 (25·5)	82 (98·8)	308 (89·5)	56·4 (33·4, 73·2)	27·5 (11·4)
**Type of device**					
Phone	16·3 (17·4)	72 (86·8)	220 (64·0)	19·0 (7·2, 31·8)	14·5 (13·1)
Laptop	9·0 (16·2)	43 (51·8)	88 (25·6)	23·9 (6·2, 51·1)	9·6 (14·6)
Tablet	3·5 (10·9)	18 (21·7)	31 (9·0)	23·4 (4·8, 54·9)	3·9 (10·3)
TV	23·5 (24·5)	70 (84·4)	192 (55·8)	37·2 (12·8, 63·1)	19·7 (16·6)
Gaming console	7·4 (17·6)	28 (33·7)	59 (17·0)	30·2 (16·5, 57·6)	6·3 (11·3)
Handheld gaming console	0·4 (2·6)	3 (3·6)	5 (1·5)	22·2 (2·2, 42·7)	0·6 (4·3)
Desktop computer	3·1 (11·4)	14 (16·9)	29 (8·4)	22·1 (9·1, 62·2)	3·0 (9·2)
Multitasking across devices	11·9 (15·0)	63 (75·9)	166 (48·3)	18·8 (8·9, 37·9)	12·9 (13·9)
E-reader	0·0 (0·0)	0 (0·0)	0·0 (0)	0·0	0·0
Other	0·1 (0·5)	2 (2·4)	4 (1·2)	3·8 (1·0, 6·6)	0·1 (0·8)
**Activity types**					
Passive^1^	36·5 (24·2)	82 (98·8)	301 (87·5)	38·1 (21·4, 59·3)	25·7 (14·2)
Interactive^2^	20·9 (22·5)	77 (92·8)	231 (67·2)	23·8 (9·2, 37·0)	17·1 (12·7)
**Screen activities**					
Watching	31·3 (24·4)	76 (91·6)	246 (71·5)	38·1 (22·1, 59·2)	23·2 (15·1)
Listening	1·7 (4·6)	24 (28·9)	33 (9·6)	7·4 (1·1, 21·2)	2·7 (7·0)
Reading	0·0 (0·3)	1 (1·2)	1 (0·3)	15·3 (15·3, 15·3)	0·1 (0·7)
Social media use	4·7 (7·9)	51 (61·5)	114 (33·1)	7·8 (2·1, 19·4)	5·5 (7·9)
Browsing	1·6 (2·2)	68 (81·9)	151 (43·9)	3·7 (0·1, 5·1)	2·3 (3·3)
Gaming	10·9 (15·6)	59 (71·1)	120 (34·9)	22·9 (10·6, 41·0)	12·1 (13·2)
Communication	3·3 (5·4)	65 (78·3)	150 (43·6)	3·7 (1·3, 7·6)	3·7 (5·8)
Multitasking within a device	5·3 (13·9)	39 (47·0)	73 (21·2)	7·7 (0·6, 24·5)	5·2 (11·5)
Educational/creative	1·3 (3·5)	31 (37·4)	46 (13·4)	4·3 (1·6, 10·5)	2·1 (5·7)
Unknown passive	1·9 (3·3)	78 (94·0)	229 (66·6)	1·3 (0·5, 3·2)	2·6 (4·6)
Unknown interactive	0·9 (0·4)	8 (9·6)	9 (2·6)	1·2 (0·8, 4·2)	0·2 (0·9)
Blocked/not in view^2^	10·1 (8·9)	80 (96·4)	263 (76·5)	10·6 (4·3, 20·0)	10·4 (10·1)

^1^ Passive activities included the total amount of time either watching, listening, reading, browsing or an unknown passive activity on a screen

^2^ Interactive activties included the total amount of either gaming, communication, multitasking, educational/creative or an unknown interactive activity on a screen

^3^ Blocked view/not in view: instances where the screen was obscured or the participant was outside the camera’s field of view

### Screen time between bedtime and shuteye time

Table 3 presents data for the time period when adolescents were in bed, but not yet trying to sleep. Overall, 57.8% of participants had screen time on 36.3% of nights for a median (25th, 75th percentiles) of 34.5 (16.5, 62.5) minutes. Although some multitasking using multiple devices still occurred in this time frame (19.3% participants, 8.7% nights, 25.7 min), the predominant device used was phones (47.0% participants, 28.8% nights, 31.0 min). Watching (32.5% participants), social media use (26.5% participants) and communication (20.5% participants) were the most common activities, with adolescents spending a median of 4.4–34.6 min on these activities. For just under half of the participants (44.6% participants), a median of 21.7 min each night could only bed coded as unknown passive, meaning it was clearly passive in nature, but unclear as to which specific activity was undertaken (i.e. watching, listening, reading). Sex differences in screen usage during this time frame were not apparent (Supplementary Table 3).

**Table 3:** Screen time between bedtime and shuteye time across device and activity types for 83 participants over a possible 344 nights

	Overall mean duration min (SD)	Number (%) of participants who had screen time	Total number (%) of nights with screen time	Median (25th, 75th percentile) duration when used, min	Mean (SD) within-person SD of screen time min
Total screen time	17·0 (26·3)	48 (57·8)	125 (36·3)	34·5 (16·5, 62·5)	15·4 (23·1)
**Type of device**					
Phone	12·6 (23·1)	39 (47·0)	99 (28·8)	31·0 (7·1, 53·5)	11·5 (20·2)
Laptop	3·3 (8·4)	14 (16·9)	23 (6·7)	46·8 (28·5, 66·8*)*	5·0 (12·5)
Tablet	0·3 (1·5)	5 (6·0)	6 (1·7)	26·2 (7·5, 29·8)	0·6 (2·9)
TV	1·2 (4·3)	11 (13·3)	14 (4·1)	22·3 (6·9, 41·0.2)	2·0 (6·7)
Gaming console	0·3 (2·3)	2 (2·4)	3 (0·9)	21·6 (14, 418)	0·5 (4·2)
Handheld gaming console	0·2 (2·0)	1 (1·2)	3 (0·9)	244 (244, 244)	0·3 (2·4)
Desktop computer	0	0	0	0	0
Multitasking across devices	2·3 (6·5)	16 (19·3)	30 (8·7)	25·7 (5·7, 34·6)	3·1 (8·5)
E-reader	0	0	0	0	0
Other	0·4 (2·9)	3 (3·6)	8 (2·3)	7·0 (2·8, 35·3)	0·5 (3·6)
**Activity types**					
Passive^1^	12·3 (21·2)	44 (53·0)	110 (32·0)	26·8 (8·7, 53·1)	12·5 (19·7**)**
Interactive^2^	2·5 (6·1)	29 (34·9)	55 (16·0)	11·6 (2·8, 21·5)	3·9 (9·3)
**Screen activities**					
Watching	5·7 (14·0)	27 (32·5)	51 (14·8)	34·6 (19.0, 47·2)	6·7 (14·0)
Listening	1·1 (5·0)	11 (13·3)	23 (6·7)	7·0 (0·9, 26·3)	1·4 (5·5)
Reading	0 (0)	0 (0)	0 (0)	0 (0)	0 (0)
Social media use	3·4 (8·7)	22 (26·5)	47 (13·7)	19·1 (11·7, 30·1)	4·5 (11·2)
Browsing	0·2 (1·5)	11 (13·3)	17 (4·9)	1·6 (0·5, 3·9)	0·5 (2·7)
Gaming	0·9 (3·5)	10 (12·1)	14 (4·1)	14·6 (1·7, 24·4)	1·5 (6·3)
Communication	0·7 (2·3)	17 (20·5)	35 (10·2)	4·4 (1·8, 9·9)	1·1 (3·4)
Multitasking within a device	0·5 (3·0)	6 (7·2)	8 (2·3)	15·9 (4·4, 21·1)	1·0 (5·8)
Educational/creative	0·0 (0·1)	3 (3·6)	3 (0·9)	2·3 (0·6, 2·8)	0·0 (0·2)
Unknown passive	5·2 (13·8)	37 (44·6)	83 (24·1)	21·7 (1·6, 22·5)	6·3 (15·1)
Unknown interactive	0·4 (2·0)	6 (7·2)	9 (2·6)	10·5 (7·3, 14·1)	0·6 (3·1)
Blocked/not in view	4·6 (19·6)	49 (59·0)	94 (27·3)	3·1 (1·3, 11·8)	4·9 (13·0)

^1^ Passive activities included the total amount of time either watching, listening, reading, browsing or an unknown passive activity on a screen

^2^ Interactive activties included the total amount of either gaming, communication, multitasking, educational/creative or an unknown interactive activity on a screen

^3^ Blocked view/not in view: instances where the screen was obscured or the participant was outside the camera’s field of view

### Screen time after shuteye time

Around one-third (32.5%) of participants had some screen time on 15.7% of nights, even after their first attempt at sleep, although the median duration was just 8.4 min. Furthermore, the within-person variability in screen time was notably lower, averaging just 3 min (Table 4). Phones were the predominant device during this period, recording a median usage of 7 min among 27% of participants, and listening was the prevailing activity (13% participants, 5.2% nights, 26 min).

**Table 4:** Screen time after first shuteye time across device and activity types for 83 participants over a possible 344 nights

	Overall mean duration min (SD)	Number (%) of participants who had screen time	Total number (%) of nights with screen time	Median (25th, 75th percentile) duration when used, min	Mean (SD) within-person SD of screen time min
Total screen time	2·6 (8.0)	27 (32·5)	54 (15.7)	8·4 (0·5, 28·6)	3·2 (7·4)
**Type of device**					
Phone	2·0 (5·6)	22 (26·5)	46 (13·4)	7·3 (0·5, 29·2)	2·8 (7·9)
Laptop	0·1 (0·3)	3 (3·6)	3 (0·9)	7·8 (1·1, 9·0)	0·1 (0·6)
Tablet	0·0 (0·0)	1 (1·2)	1 (0·3)	1·7 (1·7, 1·7)	0·0 (0·1)
TV	0·0 (0·0)	2 (2·4)	2 (0·6)	0·4 (0·3, 0·5)	0·0 (0·0)
Gaming console	0	0	0	0	0
Handheld gaming console	0·0 (0·4)	1 (1·2)	1 (0·3)	15·7 (15·7, 15·7)	0·1 (0·9)
Desktop computer	0	0	0	0	0
Multitasking across devices	0·4 (3·3)	3 (3·6)	5 (1·5)	20·2 (0·3, 39·5)	0·5 (3·5)
E-reader	0	0	0	0	0
Other	0·8 (4·7)	4 (4·8)	8 (2·3)	24·9 (21·7, 33·8)	0·8 (4·2)
**Activity types**					
Passive^1^	1·8 (5·6)	25 (30·1)	47 (13·7)	5.8 (0.5, 26.5)	2·6 (6·6)
Interactive^2^	0·6 (2·7)	10 (12·1)	18 (5·2)	4·6 (2·0, 15·7)	0·8 (3·5)
**Screen activities**					
Watching	0·1 (0·4)	5 (6·0)	5 (1·5)	1·7 (0·5, 9·5)	0·1 (0·8)
Listening	1·3 (5·3)	11 (13·3)	18 (5·2)	25·9 (12·9, 29·5)	1·8 (5·9)
Reading	0	0	0	0	0
Social media use	0·4 (2·5)	5 (6·0)	8 (2·3)	4·7 (1·2, 25·6)	0·7 (3·9)
Browsing	0	0	0	0	0
Gaming	0·2 (1·4)	3 (3·6)	4 (1·2)	15·7 (2·0, 25·1)	0·4 (2·4)
Communication	0·3 (5·4)	5 (6·0)	9 (2·6)	3·9 (3·5, 5·3)	0·4 (2·6)
Multitasking within a device	0	0	0	0	0
Educational/creative	0	0	0	0	0
Unknown passive	0·4 (2·0)	17 (20·5)	30 (8·7)	0·7 (0·5, 2·2)	0·7 (3·0)
Unknown interactive	0·1 (0·4)	4 (4·8)	6 (1·7)	2·2 (0·4, 4·5)	0·1 (0·6)
Blocked/not in view	0·8 (2·1)	27 (32·5)	43 (12·5)	2·1 (0·4, 13·9)	1·3 (3·2)

^1^ Passive activities included the total amount of time either watching, listening, reading, browsing or an unknown passive activity on a screen

2 Interactive activties included the total amount of either gaming, communication, multitasking, educational/creative or an unknown interactive activity on a screen

3 Blocked view/not in view: instances where the screen was obscured or the participant was outside the camera’s field of view

We assessed for reactivity by comparing the initial night of camera use with the subsequent three nights. No significant differences (p < 0.05) in screen time were observed in the two hours before bedtime, the time between bedtime and shut-eye time, or after shuteye time, indicating no reactivity to the initial camera exposure. When participants were asked whether the presence of the camera might have influenced their typical behaviour; 77% said they did everything they normally did, with 21% indicating they did some things differently” (2% missing data).

## Discussion

Our results indicated that almost all (99%) adolescents spent time on devices in the two hours before bed for a median duration of 56 min each night. Screen time remained prevalent (58%), even after adolescents had retired to bed for the night, with them extending their screen time for a further median duration of 35 min before attempting sleep. One-third of participants continued using their devices even after attempting sleep, although the duration was considerably shorter at a median of eight minutes. Our detailed objective analyses indicate that adolescents used a range of devices across these three evening periods, particularly non-portable televisions and phones, but also multitasking, where they used a number of devices simultaneously. They generally spent longer watching, gaming, and multitasking than on activities such as browsing, communication and social media, which were common but relatively short in duration. Although exploratory analyses suggested some sex differences in usage, numbers were small and thus should be interpreted with caution.

It is difficult to compare our findings with the literature as studies utilising objective measures of screen time that are more nuanced than simple estimates of total screen time are only just emerging [14]. Almost all research has used self-reports of screen time [22], many with unvalidated tools [13, 23], making advancing this research area challenging [3]. Our findings are in line with the results from three previous studies that have used static photo images rather than continuous video footage to measure evening screen time in similar-aged [24] or older [15, 16] adolescents. All four studies (including the current study) demonstrate that at least half the evening hours are spent on screens, predominantly phones, televisions and laptops. While direct comparisons of screen activity type are more difficult due to different categorisation in these studies, all three previous studies show large amounts of recreational screen time with watching, social networking, communicating and gaming being particularly prevalent [15, 16, 24]. Our participants spent an average of 31 min watching shows, movies, or YouTube videos before bedtime, much longer than they spent on social media at this time (5 min). While previous questionnaire-based research has indicated that young adolescents most frequently watch online videos [1], our objective data accurately quantifies these activities. This preference for streaming programs is consistent with a global shift towards digital streaming services [1, 25, 26], indicating a significant change in evening media consumption patterns worldwide.

However, none of these wearable camera studies examined screen time while in bed or after attempting sleep in adolescents, which has rarely been quantified to date. Questionnaire analyses determined that 25–75% of adolescents reported undertaking various screen activities while in bed prior to sleep but the duration of use was not provided [27]. Newer studies are utilising smartphone monitoring to assess screen time after the adolescent has gone to bed. For example, Lee et al. [12] installed an app tracker which recorded the duration of use of different forms of digital media, demonstrating that older secondary school students (mean age 18 years) spent a mean of 39 min on their devices during time awake after first going to sleep. Similarly, others have reported mean screen time of 7–11 min per hour between 10pm and 8am in 15–18 year old adolescents [9]. These figures appear considerably higher than our median total screen time after the first attempt to sleep of just 8 min, which may reflect the younger mean age of our participants (12.4 years). It is also important to emphasise that while smartphones are the main device used during these night hours, our findings highlight that other types of digital device can also contribute to screen time during the night hours. Thus, while phone tracking apps offer valuable data, they fail to capture the full range of devices and activities and have not been thoroughly validated against rigorous standards such as video-based data or direct observation [13, 14].

A particular screen behaviour of interest at present is media multitasking, typically defined as using two or more devices simultaneously. However, while multitasking is considered a normal part of modern life, especially in youth [28], accurate measurement is challenging. Current evidence suggests that self-reporting by adolescents on such complex screen use habits is unreliable, with many possibly unaware of their engagement in such behaviours [5, 29]. Our analysis demonstrates that multitasking is very common, with 47% of participants spending around 8 min using a single device to undertake two or more activities at the same time, and even higher numbers (75% of participants, median of 18.8 min) using multiple devices simultaneously in the two hours before bed. These numbers appear a little higher than those reported previously using static photo images, where 5–17% of the evening time examined involved multitasking [15, 16, 24]. However, direct comparisons are difficult as our data have not been quantified in the same manner. Our data were specifically processed so that multitasking did not inflate the total screen time estimates. However, this meant that participants could have been both multitasking within a device (e.g. YouTube minimised on the phone while scrolling through social media) while also gaming on a separate device (hence also multitasking across devices). While this was relatively infrequent, it became too complicated to have a coding schedule that had multitasking across two devices, separate from three devices and so on.

Our study has several strengths, principally around the robust design and innovative use of video footage methodology. It appears to be the first worldwide to use a reliable coding protocol to objectively quantify a spectrum of screen activities (nine types) encompassing ‘passive’, ‘interactive’, and ‘multitasking’ behaviours across a variety of devices (eight types) spanning the evening hours. This method marks a significant advancement over existing research that primarily relied on self-reported data [4, 5] or objective phone trackers that are limited to that one device [12, 13]. In particular, we were able to measure screen content, reliably categorising time spent on nine different activities of interest, which has rarely been undertaken to date [14]. We focused on the 11–14 year age group, an understudied demographic in screen time research [14]. It is important to understand evolving screen habits during this key developmental stage, which is characterised by less maturity and autonomy compared to older adolescents. Our sample, although relatively small, used a repeated measures design, which captures individual variations (within-person analysis) and affords more definitive insights into screen use habits among this age group. We had a very high completion rate (98%), which provides robustness to the data, and highlights the acceptability of this measurement technique to the families involved in our research.

Our study also has some limitations, principally regarding the labour- and time-intensive nature of video data coding. A trade-off must be made between collecting such rich and diverse data in a real-world setting with the time required to produce variables of interest. There is no doubt that the collection and analysis of such data would be prohibitive for many studies. In our study, coding was undertaken by four researchers on a part-time basis over the course of one year, a considerable research burden. Rather than using video cameras to assess usual screen time in large groups of participants, we see their value in short-term mechanistic or interventional studies in moderately sized cohorts, where accuracy of screen time measurement is particularly important. While use of video cameras will not be appropriate for all studies, machine learning techniques offer promise for the future, as evidenced by their successful application in research involving wearable image cameras for monitoring children’s dietary exposure [30]. Such development should increase the feasibility of using video cameras to measure screen time in broader sections of the population and in larger samples. However, there are also potential issues with privacy in the use of such devices in the home environment. We spent considerable time with participants and their families explaining the importance of privacy and ensured that all family members were happy with the methods used before cameras were provided. Second, we collected little data on some behaviours including reading online or engaging in educational tasks, which limited our capacity to draw conclusions about these specific types of screen use. However, it is unlikely that the diversity of screen activities captured in our study was constrained by the size of our sample, and more likely that our findings accurately depict the actual prevalence of these activities in adolescents’ real-life night-time and bedtime screen behaviours. Although a larger sample might reveal a wider spectrum of screen usage, it remains uncertain whether this would significantly alter the frequency of these rarer activities. Third, while our study was ethnically diverse including a high proportion of indigenous adolescents, the sample largely comprised parents with higher educational levels, which might limit the generalisability of our findings. Fourth, we may have overestimated television time as our coding rules classified any visible, turned-on television in the video footage as active ‘watching’ time, because it was not always clear whether the adolescent was directly watching the screen [17]. An alternative solution could be to use stationary video cameras in the living room to more clearly assess non-portable television viewing, but this would add considerable burden to both researcher and participant. Lastly, although concern has been expressed regarding potential behaviour alteration as a result of wearing the cameras, this appeared relatively uncommon in our study, whether assessed as difference in screen use across individual nights or when adolescents were directly asked whether they had changed their behaviour. We found that participants and their families were very amenable to the cameras, and rarely wanted to delete any footage before providing access to us. In instances where that occurred, it was mostly for very brief occasions (such as the adolescent forgetting to cover the camera when toileting). Such findings reiterate the acceptability of using video cameras to measure screen time in the home environment.

## Conclusion

Our findings revealed that screen use in the hours before sleep and even after attempting sleep is very common in young adolescents. Our reliable coding protocol enabled us to capture and measure screen time from video footage from wearable and stationary cameras. More work is required using objective measures of screen time to improve understanding of how screen time affects health, wellbeing and daily functioning and the feasibility and acceptability of this method for capturing objective screen time use.

## Electronic supplementary material

Below is the link to the electronic supplementary material.

Supplementary Material 1

Supplementary Material 2

## Data Availability

Data described in the manuscript, code book, and analytic code will be made available upon request pending application and approval.

## Abbreviations

BED: Bedtime Electronic Devices study
BMI: Body mass index
NZ Dep: New Zealand Deprivation Index
