# Supplementary material for: From dusk to dawn: examining how adolescents engage with digital media using objective measures of screen time in a repeated measures study
Source: Int J Behav Nutr Phys Act. 2025 Jan 7;22:4. doi: 10.1186/s12966-024-01698-0 (PMC11707906; doi:10.1186/s12966-024-01698-0)
Supplement: Supplementary file 2 — Supplementary Material 2 [file 12966_2024_1698_MOESM2_ESM.docx]

Supplementary Table 1. Length of camera footage, before bed, bedtime, shuteye time, and shuteye latency from 83 participants across 344 nights.^1^

| Variables | Median  (25th , 75th percentile) | Range  (minimum to maximum) |
| --- | --- | --- |
| Total wearable camera footage^2^, minutes | 124 (102, 148) | 19 to 191 |
| Two hours before bed camera footage^3^, minutes | 89 (68, 107) | 22 to 120 |
| After bedtime camera time^4^, minutes | 20 (9, 42) | 0 to 195 |
| Total nights | 4 (4, 4) | 2 to 6 |
| Bedtime, HH:MM | 21:33 (21:06, 22:12) | 19:01 to 01:21 |
| Shuteye time, HH:MM | 21:58 (21:29, 22:41) | 19:37 to 02:21 |
| Shuteye latency, minutes | 22 (8, 46) | 0 to 217 |

^1^ These summary variables report within-person means.

^2^ Wearable cameras were used for the before bed period.

^3^ Before bed footage is limited at two hours, with exclusions. Reasons for camera non-wear include sports, external activities, and personal care routines.

^4^ Bedtime camera footage was only coded if screens were in use during the shuteye latency period or after shuteye time, or to record shuteye time (see Supplementary Fig. 1).

Supplementary Table 2. Screen time in the two hours before bed across device and activity types by sex

|  | Males (n = 46, n = 205 nights) | | | Females (n = 35, n = 138 nights) | | |
| --- | --- | --- | --- | --- | --- | --- |
|  | Number (%) of participants who had screen time | Total number (%) of nights with screen time | Median (25th , 75th percentile) duration when used, min | Number (%) of participants who had screen time | Total number (%) of nights with screen time | Median (25th , 75th percentile) duration when used, min |
| Total screen time | 48 (100) | 187 (91.2) | 64 (42, 80) | 34 (97.1) | 120 (87.0) | 55 (33, 78) |
| **Type of device** |  |  |  |  |  |  |
| Phone | 41 (85.4) | 119 (58.0) | 15 (7, 28) | 31 (88.6) | 100 (72.5) | 23 (10, 40) |
| Laptop | 26 (54.2) | 52 (25.4) | 18 (5, 31) | 17 (48.6) | 36 (26.1) | 35 (16, 73) |
| Tablet | 9 (18.8) | 16 (7.8) | 17 (4, 63) | 9 (25.7) | 15 (10.9) | 39 (21, 45) |
| TV | 44 (91.7) | 128 (62.4) | 38 (17, 60) | 26 (74.3) | 64 (46.4) | 31 (12, 65) |
| Gaming console | 24 (50.0) | 55 (26.8) | 31 (23, 63) | 4 (11.4) | 4 (2.9) | 15 (7, 30) |
| Handheld gaming console | 3 (6.3) | 5 (2.4) | 22 (2, 43) | 0 | 0 | 0 |
| Desktop computer | 11 (22.9) | 23 (11.2) | 18 (9, 32) | 3 (8.6) | 6 (4.4) | 74 (1, 109) |
| Multitasking across devices | 40 (83.3) | 100 (48.8) | 18 (9, 36) | 23 (65.7) | 66 (47.8) | 21 (9, 38) |
| E-reader | 0 | 0 | 0 | 0 | 0 | 0 |
| Other | 2 (4.2) | 4 (2.0) | 4 (1, 7) | 0 | 0 | 0 |
| **Activity types** |  |  |  |  |  |  |
| Passive^1^ | 48 (100) | 183 (89.3) | 37 (21, 58) | 34 (97.1) | 118 (85.5) | 39 (21, 64) |
| Interactive^2^ | 47 (97.9) | 138 (67.3) | 29 (11, 47) | 29 (82.9) | 91 (65.9) | 15 (9, 26) |
| **Screen activities** |  |  |  |  |  |  |
| Watching | 46 (95.8) | 159 (77.6) | 38 (21, 59) | 30 (85.7) | 87 (63.0) | 39 (27, 62) |
| Listening | 12 (25.0) | 17 (8.3) | 5 (1, 21) | 12 (34.3) | 16 (11.6) | 11 (2, 26) |
| Reading | 1 (2.1) | 1 (0.5) | 15 | 0 |  |  |
| Social media use | 24 (50.0) | 55 (26.8) | 7 (4, 13) | 27 (77.1) | 58 (42.0) | 9 (1, 25) |
| Browsing | 41 (85.4) | 91 (44.4) | 2 (1, 5) | 27 (77.1) | 60 (43.5) | 3 (1, 5) |
| Gaming | 41 (85.4) | 91 (44.4) | 29 (11, 41) | 18 (51.4) | 29 (21.0) | 16 (7, 41) |
| Communication | 37 (77.1) | 76 (37.1) | 3 (1, 5) | 28 (80.0) | 73 (52.9) | 5 (3, 8) |
| Multitasking within a device | 26 (54.2) | 52 (25.4) | 9 (1, 39) | 11 (31.4) | 19 (13.8) | 1 (0.3, 15) |
| Educational/creative | 20 (41.7) | 30 (14.6) | 4 (2, 9) | 11 (31.4) | 16 (11.6) | 7 (1, 46) |
| Unknown passive | 45 (93.8) | 136 (66.3) | 1 (1, 3) | 33 (94.3) | 93 (67.4) | 1 (0.4, 2) |
| Unknown interactive | 4 (8.3) | 4 (2.0) | 1 (1, 2) | 4 (11.4) | 5 (3.6) | 3 (1, 9) |
| Blocked/not in view^2^ | 46 (95.8) | 147 (71.7) | 9 (3, 18) | 34 (97.1) | 116 (84.1) | 13 (6, 21) |

^1^ Passive activities included the total amount of time either watching, listening, reading, browsing or an unknown passive activity on a screen.
^2^ Interactive activties included the total amount of either gaming, communication, multitasking, educational/creative or an unknown interactive activity on a screen.
^3^ Blocked view/not in view: instances where the screen was obscured or the participant was outside the camera’s field of view

SupplementaryTable 3. Screen time between bedtime and shuteye time across device and activity types by sex

|  | Boys (n = 46, n = 205 nights) | | | Girls (n = 35, n = 138 nights) | | |
| --- | --- | --- | --- | --- | --- | --- |
|  | Number (%) of participants who had screen time | Total number (%) of nights with screen time | Median (25th , 75th percentile) duration when used, min | Number (%) of participants who had screen time | Total number (%) of nights with screen time | Median (25th , 75th percentile) duration when used, min |
| Total screen time | 27 (56.3) | 72 (35.1) | 28 (6, 79) | 21 (60.0) | 53 (38.4) | 35 (23, 48) |
| **Type of device** |  |  |  |  |  |  |
| Phone | 22 (45.8) | 54 (26.3) | 24 (3, 54) | 17 (48.6) | 45 (32.6) | 36 (17, 43) |
| Laptop | 8 (16.7) | 12 (5.9) | 54 (29, 79) | 6 (17.1) | 11 (8.0) | 40 (27, 67) |
| Tablet | 2 (4.2) | 3 (1.5) | 4 (1, 7) | 3 (8.6) | 3 (2.2) | 30 (26, 36) |
| TV | 8 (16.7) | 11 (5.4) | 29 (13, 50) | 3 (8.6) | 3 (20.2) | 21 (0.03, 23) |
| Gaming console | 2 (4.2) | 3 (1.5) | 22 (1, 42) | 0 | 0 | 0 |
| Handheld gaming console | 1 (2.1) | 3 (1.5) | 24 | 0 | 0 | 0 |
| Desktop computer | 0 | 0 | 0 | 0 | 0 | 0 |
| Multitasking across devices | 9 (18.8) | 18 (8.8) | 31 (3, 35) | 7 (20.0) | 12 (8.7) | 20 (8, 34) |
| E-reader | 0 |  |  | 0 |  |  |
| Other | 2 (4.2) | 6 (2.9) | 19 (3, 35) | 1 (2.9) | 2 (1.4) | 7 (7, 7) |
| **Activity types** |  |  |  |  |  |  |
| Passive^1^ | 25 (52.1) | 64 (31.2) | 28 (8, 65) | 19 (54.3) | 46 (33.3) | 26 (22, 95) |
| Interactive^2^ | 16 (33.3) | 32 (15.6) | 4 (2, 19) | 13 (37.1) | 23 (16.7) | 20 (12, 30) |
| **Screen activities** |  |  |  |  |  |  |
| Watching | 16 (33.3) | 33 (16.1) | 32 (12, 45) | 11 (31.4) | 18 (13.0) | 37 (21, 47) |
| Listening | 4 (8.3) | 8 (3.9) | 7 (0.4, 24) | 7 (20.0) | 15 (10.9) | 7 (1, 26) |
| Reading | 0 |  |  | 0 |  |  |
| Social media use | 12 (25.0) | 28 (13.7) | 19 (9, 29) | 10 (28.6) | 19 (13.8) | 19 (12, 30) |
| Browsing | 7 (14.6) | 10 (4.9) | 2 (0.4, 4) | 4 (11.4) | 7 (5.1) | 1 (1, 9) |
| Gaming | 8 (16.7) | 12 (5.9) | 11 (2, 29) | 2 (5.7) | 2 (1.4) | 15 (14, 16) |
| Communication | 7 (14.6) | 16 (7.8) | 2 (1, 5) | 10 (28.6) | 19 (13.8) | 8 (3, 20) |
| Multitasking within a device | 3 (6.3) | 4 (2.0) | 4 (0.2, 19) | 3 (8.6) | 4 (2.9) | 21 (13, 99) |
| Educational/creative | 3 (6.3) | 3 (1.5) | 2 (1, 3) | 0 | 0 | 0 |
| Unknown passive | 23 (47.9) | 53 (25.9) | 8 (1, 25) | 14 (40.0) | 30 (21.7) | 6 (2, 14) |
| Unknown interactive | 2 (4.2) | 2 (1.0) | 11 (8, 13) | 4 (11.4) | 7 (5.1) | 11 (4, 30) |
| Blocked/not in view^2^ | 29 (60.4) | 61 (29.8) | 3 (2, 12) | 20 (57.1) | 33 (23.9) | 3 (1, 15) |

^1^ Passive activities included the total amount of time either watching, listening, reading, browsing or an unknown passive activity on a screen.
^2^ Interactive activties included the total amount of either gaming, communication, multitasking, educational/creative or an unknown interactive activity on a screen.
^3^ Blocked view/not in view: instances where the screen was obscured or the participant was outside the camera’s field of view

Supplementary Figure 1. Video camera study timeline: two hours before bed to after shuteye time.


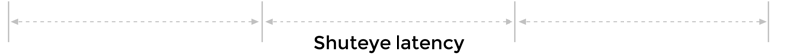


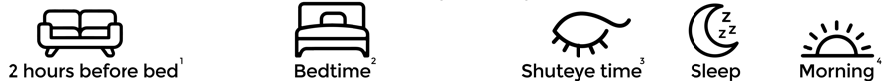


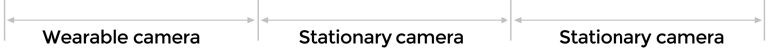


^1^Wearable cameras may not have been worn for the full two hours before bed due to sport, activity outside the house, bathing, and dressing.

^2^Bedtime (first point participant gets into bed for the night).

^3^Shuteye time (first point where participant attempts to fall asleep for the night).

^4^Morning (video cameras turned off upon waking in the morning).
